# Predictors of Low Back Pain Risk Among Farmers in Rural Communities of Loja, Ecuador

**DOI:** 10.3390/ijerph22060885

**Published:** 2025-05-31

**Authors:** Isabel Masson Palacios, Israel Vinueza-Fernandez, Samuel-Olegario Iñiguez-Jiminez, Mario J. Grijalva, Benjamin R. Bates

**Affiliations:** 1 Escuela de Fisioterapia, Facultad de Ciencias Médicas, de la Salud y la Vida, Universidad Internacional del Ecuador, Quito 170150, Ecuador; mymassonpa@uide.edu.ec; 2 Facultad de Enfermería, Centro de Investigación para la Salud en América Latina (CISeAL), Pontifica Universidad Católica del Ecuador, Quito 170150, Ecuador; iavinuezaf@puce.edu.ec; 3 Faculty of Healthy and Well-Being, School of Physical Therapy, Centro de Investigación para la Salud en América Latina (CISeAL), Pontifica Universidad Católica del Ecuador, Quito 170150, Ecuador; soiniguez@puce.edu.ec; 4 Infectious and Tropical Disease Institute, Department of Biomedical Sciences, Heritage College of Osteopathic Medicine, Ohio University, Athens, OH 45701, USA; grijalva@ohio.edu; 5 School of Communication Studies, Scripps College of Communication, Ohio University, Athens, OH 45701, USA; 6 Centro de Investigación para la Salud en América Latina (CISeAL), Pontifica Universidad Católica del Ecuador, Quito 170150, Ecuador

**Keywords:** low back pain, risk factors, prediction model, farmers, 12-month prevalence

## Abstract

Background: Low back pain (LBP) and musculoskeletal disorders are highly prevalent among agricultural workers. However, there is limited epidemiological evidence from rural regions of Ecuador, where working and living conditions may differ substantially from those in other settings. This study aimed to identify predictors of LBP among farmers in rural Ecuador to inform locally relevant prevention strategies. Methods: Participants aged 30 to 60 years (*n* = 103) were recruited through a traveling health clinic. Participants were assessed with behavioral and sociodemographic self-report questionnaires and anthropometric measurements. Low back pain (LBP) was assessed using the Standardized Nordic Musculoskeletal Questionnaire, which asked about symptoms experienced in the past 12 months. Bivariate (Chi-square and Fisher exact tests) and multivariate (binary logistic regression) analyses were conducted to explore associations between risk factors and LBP in individuals aged 30 to 60 years. Results: LBP was highly prevalent, affecting 78.6% of participants. Behavioral patterns were mixed, with low rates of smoking and moderate alcohol and coffee consumption associated with LBP. A normal body mass index (BMI) was observed in 66% of the sample, and over half reported stable mood and good self-perceived health. In the binary logistic regression analysis, only education level significantly predicted LBP, with secondary education acting as a protective factor. Conclusions: While lower back pain was widespread in the population studied, most risk factors that were analyzed were not significantly associated with its presence.

## 1. Introduction

Low back pain (LBP) and musculoskeletal disorders (MSDs) are major occupational health concerns among agricultural workers worldwide, leading to significant physical impairment, reduced productivity, and socioeconomic burden, often exacerbated by negative effects on mental health [1]. These conditions are particularly concerning in low- and middle-income countries (LMICs), where agricultural labor is more physically demanding, access to health services is limited, and informal work arrangements are common [2].

In Latin America, and especially in rural Ecuador, agriculture remains a primary source of livelihood, yet there is limited epidemiological data on the burden of LBP and MSDs in these populations. Available studies suggest that Latin American agricultural workers may report lower prevalence rates of LBP (11–20%), but these figures may underestimate the true burden due to underreporting, differences in recall periods, and a lack of standardized surveillance systems [3,4]. In this context, biomechanical risk factors such as improper posture, frequent lifting of heavy objects, repetitive movements, and prolonged exposure to vibrations from machinery are primary contributors to LBP and MSDs in agricultural settings [5,6].

Estimates from high-income countries show that LBP affects 37% of agricultural workers in the United States and Ireland, based on 12-month prevalence data [5,6]. In Sweden, 12-month prevalence has been reported to be as high as 47% [7]. In LMICs, prevalence rates vary widely, ranging from 21% [8] to nearly two-thirds (63%) of agricultural workers reporting LBP within the past year [9]. Within LMICs, the highest prevalence has been observed in Asia (56–64%, mostly 12-month prevalence) [10,11,12], and Africa (57–72%, though some studies report lifetime prevalence) [13,14].

LBP in this population rarely occurs in isolation and is often associated with other musculoskeletal disorders (MSDs), chronic pain conditions, and functional disability [5,15,16]. For example, among Irish farmers, 37% identified back pain as their most significant health issue in the previous year [5]. Moreover, studies on farmers show that 73% report experiencing LBP, making it the most prevalent MSD, while other musculoskeletal complaints—such as elbow (28%), wrist, hip, knee, and foot pain—range from 40% to 56%, although the recall periods are not always specified [17]. These findings align with previous research identifying LBP as the most frequently reported MSD, followed by pain in the shoulders, knees, and neck [6]. The recurrent nature of LBP, with acute episodes often lasting a week or more, highlights the ongoing burden in agricultural workers [15].

Several studies report that approximately one-third of agricultural workers experience LBP, with neck and shoulder pain affecting about 30.8%, and pain in the elbow, wrist, or hand reported by 21.6% of workers. The average duration of work incapacity due to MSD-related discomfort is around two weeks [18]. Furthermore, 15.9% of individuals with MSDs reported an inability to perform agricultural duties or the need for medical consultation, primarily due to LBP, although recall periods vary across studies [5].

The development of MSDs is closely linked to the type of agricultural work performed and seasonal variations. For instance, during the planting season, knee pain is most prevalent (19.6%), followed by LBP (16.1%), while wrist, shoulder, and hip pain occur less frequently. In contrast, during the harvest season, MSD prevalence significantly increases in the shoulder region due to repetitive and strenuous activities [19].

Ergonomic risk factors correlate with anatomical MSD locations; a Korean study found that livestock workers had higher risks for neck and upper extremity MSDs, while irrigation workers experienced more back-related MSDs. Forced neck postures, repetitive hand or wrist movements, prolonged flexion or torsion of the upper body, and lifting loads over 10 kg were key contributors [20].

Moreover, tasks such as rice planting and seedling transplantation, which involve uncomfortable postures, repetitive movements, and prolonged standing, were strongly associated with upper and lower extremity pain, sometimes more so than manual material handling [11].

Beyond occupational risks, individual characteristics such as age and body composition influence MSD prevalence. In Ecuador, 38% of farmers are overweight or obese. Younger farmers (18–54 years) report higher neck pain prevalence compared to older farmers (58.96% vs. 48.65%, *p* = 0.043), while knee pain is more common among older farmers (45.61% vs. 60.27%, *p* = 0.004) [21]. LBP prevalence generally increases with age due to prolonged exposure and age-related changes in pain perception [17].

This study presents ECU-TME, a cross-sectional study of adult agricultural workers residing in the communities of Calvas and Gonzanamá in Loja Province, Ecuador, a region in the southern part of the country near the border with Peru. These rural communities are characterized by unique working conditions and demographic profiles, including high-altitude farming (1500–2500 m above sea level), limited mechanization that increases physical labor demands, an aging workforce, and restricted access to occupational health services.

Given the complexity of LBP and MSDs and considering the specific socioeconomic and cultural context of rural Ecuador, this study focuses on several key domains that may influence LBP risk. These include sociodemographic characteristics (e.g., age, sex, education), lifestyle factors (e.g., physical activity, smoking), anthropometric indicators (e.g., body mass index, obesity), and self-rated health and mood, which have been highlighted in previous studies as important contributors to musculoskeletal health.

We need to study Ecuador because it is classified as a lower-middle-income country, facing various socioeconomic challenges that impact its rural populations [21], in rural areas, and a significant proportion of the population is engaged in agricultural labor, which remains a primary source of livelihood and economic activity [22,23]. However, these rural communities often experience disparities in access to economic opportunities, social services, and political representation, which exacerbate their vulnerability and limit their capacity to address health and unemployment [24]. This context underscores the importance of understanding the occupational health challenges faced by agricultural workers in Ecuador. Therefore, we ask the following research questions:

- RQ1: What is the 12-month prevalence of lower back pain (LBP) among agricultural workers in rural communities of Loja Province, Ecuador?
- RQ2: What occupational exposures, ergonomic factors, and individual health characteristics are associated with the 12-month prevalence of LBP among agricultural workers in rural Loja Province?

## 2. Materials and Methods

### 2.1. Study Population and Data Collection

The sample size was determined using a 95% confidence level and a 10% margin of error, based on the combined population of 38,289 inhabitants in Calvas and Gonzanamá as of 2022. Population data were sourced from the Instituto Nacional de Estadística y Censos (INEC) [25].

To answer the research questions, a convenience sample was used. The sample was collected from individuals who attended a series of health evaluation fairs held in two rural counties. In June 2023, project personnel, Ministry of Public Health of Ecuador (MOH) staff, and community leaders in Calvas and Gonzanamá counties, Loja Province advertised a free health evaluation fair. Calvas and Gonzanamá were selected because they have the highest rural population density in the Loja Province. In addition, these communities have limited access to healthcare services, and their primary economic activity is agriculture.

Following promotion of the health evaluation fairs, during two weeks in June–July 2023, a set of mobile stations were established at rotating locations for interested individuals to attend. These locations were schools, gymnasiums, and other community centers in rural communities. The evaluation area was consistent across all locations, and the same evaluation personnel conducted the assessments. All staff members received prior training to ensure standardized application of the evaluation instrument. Community members either walked to the health fair (if they lived within 2 km) or were given rides by project staff (if they lived farther than 2 km from the site of the fair). For this study of LBP, individuals aged 30 to 60 years, able to understand Spanish, and not having a physical or mental disability registered by MOH were invited to participate in the study. At the first station at the health evaluation fair, individuals were informed about the research associated with the study, eligible individuals were invited to participate, and those who agreed were invited to sign an informed consent form approved by the research ethics committee of an Ecuadorian university (CEISH-461-2023). After agreeing to participate or declining, the individual then proceeded to other stations at the fair. Although all attendees at the fair received assessments at all stations, only those who consented to the research had their data recorded for this study.

### 2.2. Clinical Assessments

The protocol was designed to assess the predictors of LBP among agricultural workers. Participants were administered questionaries, and their weight and height were obtained. Participants were also asked about musculoskeletal disorders with the Nordic Musculoskeletal Questionnaire (NMQ), which was defined using specific items about LBP if the participants reported having experienced pain or discomfort in the lower back region within the past 12 months. Three domains of predictors of LBP were assessed (see Table 1 for a list of domains and domain-specific topics evaluated in the study).

The first domain was sociodemographic and lifestyle measures. Trained interviewers asked participants a series of questions about their sociodemographic characteristics, including age (years), sex (M/F), self-identified ethnicity, and marital status. Information about individuals’ living arrangements, number of household members, employment status, number of hours worked per week, average family income, and number of children was also collected (see Table 1). Additionally, participants were asked a set of questions about their lifestyle. For instance, they were asked about their tobacco, coffee, and alcohol consumption, and the number of meals a day (see Table 1). Standard demographic items such as civil status, education, ethnicity, living arrangements, and total household income were included to allow potential comparisons to other populations with LBP. Chronic disease and disability are factors often associated with LBP, while several occupational variables may put people at risk of LBP, including occupation, job type, work posture, crops produced, years of work, labor statis, and hours worked per day. A series of behavioral factors may influence the experience of LBP, such as tobacco use, alcohol use, and coffee consumption. Finally, overall health and overall mood are associated with other forms of wellbeing and, potentially, with LBP.

In the second domain, anthropometric measurements were assessed using calibrated SECA clinical-grade equipment. The personnel conducting the evaluations had been previously trained in measuring weight (without shoes, in kilograms) and height (in centimeters). The assessment procedures strictly adhered to standardized protocols. Height and weight were used to calculate body mass index. Ecuador’s standard cut-offs were used to classify individuals as underweight (BMI < 18.5), normal weight (BMI 18.5–24.9), or overweight (BMI ≥ 25) [26]

The final domain was self-rated health and self-rated mood. Self-rated health was assessed with a single item: “In general, would you say your health is” with the response items ranging from “excellent” to “poor”. This item is a valid and broadly accepted global measure of an individual’s perception of their health [27]. Self-rated mood was assessed with a single item: “In general, would you say your mood is” with response options ranging from “excellent” to “poor”. This item is also a valid and broadly accepted global measure of an individual’s perception of the mental wellbeing [28]. However, despite their validity, these single-item measures have limitations in capturing the full complexity and nuances of each construct.

### 2.3. Statistical Analysis

Descriptive statistics for demographic characteristics, anthropomorphic measures, and the self-report measures are presented in terms of relative (%) and absolute (N) values. All variables were recorded as nominal or ordinal variables. Associations between these variables and presence or absence of LBP were assessed with Chi-square tests. If cell counts were <5, Fisher’s exact tests were used. For these tests of association, for large effects where α = 0.05 and β = 0.80, with the largest degree of freedom in the contingency tables set at 5, a minimum sample size of 52 was required [29]. The level of statistical significance was set at *p* < 0.05. Cramer’s v was used to assess effect size.

The results of the Chi-square tests determined which potential predictors were entered into a binary logistic regression model. Following the Chi-square tests, statistically significant associations were entered into the binary logistic regression using the simultaneous entry method. Binary logistic regression was chosen both because the dependent variable of presence/absence of LBP is binary, but also because logistic regression is a mathematical model control for confounders. Simultaneous entry was chosen because we did not have a hypothesis as to which variables were most important and the number of predictors (3) was small. For a logistic regression, the minimum number of cases to include is N = 10 × k/*p*, where k is the number of covariates to include in the model and p is the proportion of positive cases in the population [30]. Given three predictors in the binary logistic regression model (see below: education, overall health, overall mood) and the proportion of persons experiencing LBP in the population is 0.79, a minimum sample size for the binary logistic regression was 24 persons (N = 23.7 = 10 × (3 × 0.21). Common rules of thumb suggest that there should be 10 cases for each predictor, or N = 30 [31]. However, following Long’s [32] recommendation, when either the power calculation or the rule of thumb results in fewer than 100 cases, the minimum sample size should be increased to 100. The strength of association was expressed as odds ratios (ORs) with 95% confidence intervals (CIs).

All statistical analyses were performed using SPSS Statistics for Windows, version 29.0. A significance level of *p* < 0.05 was adopted for all tests.

## 3. Results

### 3.1. Subject Characteristics

The present study was conducted in rural communities in the province of Loja, Ecuador. One hundred and three individuals agreed to participate. Of these participants, about half (*n* = 51, 49.5%) were men and half (*n* = 52, 50.5%) were women. The average age was 46.51 +/− 9.76. Nearly all (94.2%) identified as mestizo (i.e., mixed Spanish and indigenous heritage). About two-thirds of the participants (*n* = 64, 62.1%) were married, and most (*n* = 85, 82.5%) of them lived with their families. The average number of children was 3.52 +/− 2.60, and the average household size was 4.19 +/− 2.179 people (see Table 2 for full demographics).

The participants were generally economically marginalized. More than half of the participants (*n* = 58, 56.3%) were informally employed, indicating under-the-table day labor or working on one’s own farm. A plurality (*n* = 43, 41.7%) worked more than 40 h per week. Despite long working hours, 43 persons (41.7% of the population) reported earning between USD 0 and USD 100 per month, an income level that places them below Ecuador’s poverty line (Table 2).

Lower back pain was very common in our sample, with 81 participants (78.6%) reporting current LBP. The participants reported mixed behavioral risk factors. Most participants were never smokers (*n* = 87, 84.5%). Participants drank alcohol at least occasionally (*n* = 77, 74.8), and almost all drank coffee at least occasionally (*n* = 96, 932%). Most participants reported consuming low-alcohol beverages such as beer or wine sporadically (72.8%), while 60.2% consumed high-alcohol beverages. About half (54.4%) maintained a regular schedule for their meals, and 75.7% consumed three meals a day or less. About two-thirds of the participants (66%) had a normal body mass index, while 17% were classified as each being overweight or underweight. In terms of self-perceived health, 36.9% claimed their health to be “acceptable”. Regarding chronic diseases, 68.0% did not report any pathologies. As for mood state, 51.5% reported a normal mood. Overall, the participants appear to be largely reflective of the 30–60 year old populations of Calvas and Gonzanama counties.

### 3.2. Predictors of Low Back Pain

#### 3.2.1. Bivariate Analyses

We began with Chi-square tests to explore associations between candidate predictor variables and LBP. As indicated in Table 2, most candidate variables were not associated with the presence or absence of LBP. Specifically, only level of education, self-reported health, and self-reported mood met conventional thresholds of statistical significance to be considered potential predictors of LBP. There was a weak association between mood and LBP (*v* = 0.17), and moderate associations between LBP and level of education (*v* = 0.307) and self-reported overall health (*v* = 0.246).

#### 3.2.2. Multivariate Analysis

These predictors were then entered into a binary logistic regression model to predict LBP. The binary logistic regression analysis showed a statistically significant association between risk factors and LBP (Table 3). Education level presented as a protective factor related to LBP. The binary logistic regression revealed that persons with a secondary level of education were less likely to experience LBP than persons with a primary level of education (OR 0.295 (0.092, 0.950), *p* = 0.041). Specifically, persons with a secondary education experienced LBP at about 1/3 the rate of those who had only a primary education. Additional postsecondary education, however, was not further protective (OR 0.598 (0.074, 4.813), *p* = 0.639). Although the initial Chi-square analysis indicated that presence/absence of LBP was associated with self-reported health and self-reported mood, the binary logistic regression failed to reveal a statistically significant association. Rather, since all individuals with poor self-reported health and self-reported mood reported LBP, it is likely that the relationship is not logarithmic but may simply be linear.

## 4. Discussion

Firstly, this study focuses on the rural communities in the province of Loja, Ecuador, where the main occupation is agriculture. Agricultural activity in this area includes the production of sugarcane, coffee, cereals, and other products such as corn, beans, peas, wheat, barley, cassava, and carrots [33]. This dependence on the agricultural workforce in an environment of potentially restricted resources could lead to specific vulnerabilities in terms of collective health.

Low back pain is the most prevalent MSD among the working population globally [34,35,36]. Research conducted in LMICs indicates a higher prevalence of low back pain among farmers compared to developed countries [37]. In Ecuador, a study conducted in Cuenca reported a prevalence of 9.3% of low back pain in the general population, with residents of rural areas reporting higher musculoskeletal pain [38]. That study agreed with other research in Ecuador showing that the prevalence rates of LBP among agricultural workers are high, ranging between 75% and 94% of rural people in Ecuador reporting a history of LBP at some point in their lives [19,26]. Thus, it is not surprising that our study found that more than three-quarters of the participants (78.6%) reported current LBP.

Frequently, rural workers are at a higher risk of suffering from low back pain due to the nature of their work and the high demands of physical effort [39]. The prevalence of LBP in our sample exceeds the 28.52% (IC del 95% = 10.91–50.33) reported in Latin American agricultural workers in meta-analyses [9,40]. Within the meta-analyses of LBP among laborers in Latin America, 14 of the 28 studies in one study [9] and 3 of the 4 studies in the other study [40] were conducted in Brazil. Moreover, the Brazilian sample sizes tended to be larger than those from other Latin American countries, weighting the results substantially toward a Brazilian experience. The different rates of LBP may be due to the greater rates of mechanization and automation of agricultural labor in Brazil as compared to Ecuador [41] as well as topological differences between the two countries [42]. That is, Brazilian agricultural workers are more likely to plant, harvest, and process crops with machines on relatively flat land while Ecuadorian agricultural workers are more likely to manually plant, harvest, and process crops on steep hillsides. Because Ecuadorian agricultural workers are more likely use human labor for farming tasks, with minimal or no use or machinery, they are required to engage in repetitive and strenuous tasks, while in uncomfortable positions, and often under heavy loads; therefore, it should not be surprising that they experience high rates of LBP as compared to Brazilian agricultural workers. This chronic pathology can cause significant direct and indirect costs for individuals and the healthcare systems of Ecuador. In Ecuador, LBP is the main cause of activity restriction in individuals under 45 years old and the third most common cause of activity restriction in people aged 45 and older, in addition to being the most common disease in people aged 65 and older [38].

Although we did not find statistically significant associations among most sociodemographic factors and LBP, our sample reveals some important insights about changes in the demography of rural agriculture in Ecuador. Although a larger sample size potentially could have revealed weak or moderate associations, we found no large effects of these factors.

In addition, in the present study, as part of the selection criteria, only persons between 31 and 60 years were invited to participate. The average age of farmers in Ecuador is 47 years. However, in certain traditional crops (such as corn and potatoes), the average age can exceed 50 years [43]. Most farmers are between 45 and 64 years old, which could suggest an aging in this industry due to a lower generational transition, the migration of young people to cities, a lack of access to technology, or the absence of incentives for future generations [44].

Regarding gender distribution, our figures show that in these two counties, about half of the rural agricultural workers are women (50.5%), a slight increase in the presence of women in this sector. This could suggest that women are playing a crucial role in the economy of Loja Province, reflecting a significant shift in the agricultural labor dynamics, taking on an imperative role in managing small businesses, and collaborating in crop production and livestock activities [40,45,46]. However, conventional gender norms can restrict women’s access to resources and training in the agricultural sector, which could impact their ability to incorporate healthy agricultural practices [47]. LBP, which is common among women in the study, can adversely impact their ability to carry out agricultural tasks, many of which require intense physical effort and prolonged postures. This circumstance could result in a reduction in productivity and an increase in absenteeism, impacting not only the personal wellbeing of the employees but also the economy of the family and the community, where their role is essential. Therefore, this relevant finding can be considered for the research of gender-sensitive public policies.

Moreover, in terms of ethnicity, a considerable portion of farmers in our study identified themselves as mestizos. It may be that, in this region of Ecuador, the agricultural worker population is primarily mestizo, excluding Indigenous people, “White” Ecuadorians (persons of only Spanish heritage), Afro-Ecuadorians, and Montubios (persons of mixed African, European, and Indigenous heritage) from this sector. Regarding the level of education, a study in the country noted that a high percentage of farmers have a primary education level [48]. Our study found similar educational attainment. Since low educational attainment was predictive of LBP, supporting stronger education overall may also serve to protect agricultural workers from LBP.

Moreover, in rural communities, restricted access to healthcare and lower resources for prevention are attributed to poverty and low socioeconomic status [49,50]. Our study found that many of our participants were in a condition of economic vulnerability. More than half had informal jobs, and a considerable percentage subsisted below the country’s poverty line, with monthly incomes not exceeding USD 100. Given these high rates of economic vulnerability, it is not surprising that there is also broad experience of MSD across the communities.

Tasks such as planting, weeding, and harvesting crops frequently require farmers to be in bent positions for long periods of time. Our analysis indicated that the rates of lower back pain are high in all work positions (66.7–83.3%). This indicates a wide prevalence regardless of posture. It is important to highlight that various studies indicate that the implementation of positions such as trunk flexion, twisting, kneeling, and extended trunk flexion are significant contributions [9,51,52,53,54]. This could be the result of applying significant tension to the lumbar spine and could cause fatigue and pain as a defense mechanism against prolonged posture over time [51].

Although smoking is frequently associated with an increased risk of low back pain in agricultural workers [9,40,55], it is also common for obesity to be an additional risk factor for musculoskeletal disorders [9,55]. In this study, no relevant links were found between low back pain and smoking, alcohol, coffee, or BMI (all *p*-values > 0.05). This indicates that other unassessed factors could play a more significant role.

On the other hand, it has been reported that intense physical effort, long work periods, monotony, and task repetition are identified as relevant psychosocial factors linked to musculoskeletal disorders [54,56]. In this regard, the results of the present research indicate that self-perception of health plays a crucial role, as users who consider their health as “poor” or “acceptable” present a higher risk of LBP (84–100%) compared to those who categorize it as “good/very good” (33–65%), suggesting a relationship between LBP and perceived poor general health, possibly due to chronic conditions, stress, or physical restrictions. Additionally, higher levels of stress are linked to an increased risk of lumbar pain [57], and the results of the present study regarding self-reported low mood correlate with higher LBP (100% in “poor” vs. 67.5% in “good”). It is likely that there is a bidirectional influence between chronic pain and mood, where a low mood can cause muscle tension and alterations in pain perception, favoring its onset or persistence [58].

Finally, in Ecuador, the primary education system is free, mandatory, and lasts for 6 years (from first to sixth grade, for children approximately 5 to 11 years old) [28]. This explains that, in the presented results, the majority of the participants have primary education. In this way, having secondary education (compared to only primary) reduces the risk of LBP by 70.5% (protective effect). Therefore, basic education is considered a relevant predictor linked to a reduced risk of LBP. A lower level of education could be linked to reduced awareness of ergonomic risks and preventive strategies for lower back pain [29]. This could be based on the fact that individuals with more years of education generally have more efficient access to and understanding of health-related information, including occupational health, fostering the development of skills to obtain and use knowledge, which promotes the adoption of correct postures, proper lifting techniques, and other preventive measures and ergonomics [59]. Additionally, higher education is often linked to jobs that are less physically demanding [60]. In summary, a broader education can promote a more robust culture of prevention and self-care.

## 5. Conclusions

This study concludes that no significant relationship was found between the analyzed risk factors and the presence of low back pain. Therefore, these factors cannot be considered reliable predictors of low back pain in the studied population.

It is important for future research to focus on working conditions and other unexamined risk factors in order to establish a stronger foundation for the prevention of musculoskeletal injuries, particularly in communities with limited access to healthcare.

### Limitations

This study has several limitations that should be acknowledged. First, as an observational study without follow-up, it is not possible to establish causal relationships. Second, the findings should be interpreted with caution, as this research does not allow for the prediction of low back pain (LBP) among agricultural farmers. Third, data were collected using a self-report questionnaire, which may introduce response bias due to potential over- or underestimation of LBP. Fourth, the study was conducted in communities from only two counties, and the relatively small sample size may limit the representativeness of the results. Therefore, our findings cannot be generalized to all rural communities. Despite these limitations, this study is the first to evaluate predictors of LBP among farmers in rural communities, providing valuable insights for future research.

## Figures and Tables

**Table 1:** Anthropometric measures and health.

Health Domain	Assessment
Sociodemographic and lifestyle measures	Marital status Education Ethnicity Chronic disease Disability Occupational background Job type Main work posture Main products produced Years of work Laboral status Hours worked per day Living arrangement Household income Tobacco, coffee and alcohol consumption Number of meals a day Defined meals schedule
Anthropometric measures and health	Weight, height, body mass index (BMI)
Mental health and cognition	Perception of health status Perception of mood state

**Table 2:** Sociodemographic and individual characteristics associated with LBP among farmers.

Variable	N (%) 103	Without LBP	with LBP	Chi-Square Value	*p*	*v*
Sex				0.184	0.668	0.042
Male	51 (49.5)	10 (19.6)	41 (80.4)			
Female	52 (50.5)	12 (23.1)	40 (76.9)			
Age				1.946	0.584	0.143
21–30	3 (3.2)	0 (0.0)	3 (100.0)			
31–40	29 (30.5)	8 (27.6)	21 (72.4)			
41–50	19 (20.0)	5 (26.3)	14 (73.7)			
51–60	44 (46.3)	8 (18.2)	36 (81.8)			
Civil Status				1.412	0.842	0.117
Single	18 (17.5)	4 (22.2)	14 (77.8)			
Married	64 (62.1)	14 (21.9)	50 (78.1)			
Partnered	11 (10.7)	3 (13.6)	8 (72.7)			
Divorced/Separated	6 (5.8)	1 (16.7)	5 (83.3)			
Widowed	4 (3.9)	0 (0.0)	4 (100.0)			
Ethnicity				5.045	0.283	n.c.
Mestizo	97 (94.2)	21 (21.6)	76 (78.4)			
Afrocecuadorian	3 (2.9)	0 (0.0)	43 (100.0)			
Montubio	1 (1.0)	1 (100.0)	0 (0.00)			
Blanco	1 (1.0)	0 (0.0)	1 (100.0)			
Other	1 (1.0)	0 (0.0)	1 (100.0)			
Household				0.285	0.867	0.053
Lives alone	7 (6.8)	2 (28.6)	5 (71.4)			
With family	85 (82.5)	18 (21.2)	67 (78.8)			
With partner	11 (10.7)	2 (18.2)	9 (81.8)			
Number of children				3.255	0.516	0.178
0	8 (7.8)	3 (37.5)	5 (62.5)			
1	11 (10.7)	2 (18.2)	9 (81.8)			
2	23 (22.3)	4 (17.4)	19 (82.6)			
3	19 18.4)	6 (31.6)	13 (68.4)			
4 or more	42 (40.8)	7 (16.7)	35 (83.3)			
Education				9.724	**0.008**	**0.307**
Primary	62 (60.2)	7 (11.3)	55 (88.7)			
Secondary	34 (33.0)	13 (38.2)	21 (61.8)			
Post-secondary	7 (6.8)	2 (28.6)	5 (71.4)			
Employment				2.869	0.412	0.167
Formal	25 (24.3)	7 (28.0)	18 (72.0)			
Informal	58 (56.3)	12 (20.7)	46 (79.3)			
Occasional	14 (13.6)	1 (7.1)	13 (92.9)			
Does not work	6 (5.8)	2 (33.3)	4 (66.7)			
No. years worked				2.124	0.711	0.146
1–10	17 (17.0)	3 (17.6)	14 (82.4)			
11–20	33 (33.0)	7 (21.2)	26 (78.8)			
21–30	16 (16.0)	5 (31.3)	11 (68.8)			
31–40	17 (17.0)	2 (11.8)	15 (88.2)			
41 or more	17 (17.0)	3 (17.6)	14 (82.4)			
Hours worked/week				5.933	0.204	0.241
1–10	9 (8.8)	3 (33.3)	6 (66.7)			
11–20	8 (7.8)	0 (0.0)	8 (100.0)			
21–30	10 (9.8)	3 (30.0)	7 (70.0)			
31–40	32 (31.4)	4 (12.5)	28 (87.5)			
40 or more	43 (42.2)	12 (27.9)	31 (72.1)			
Income/month				6.984	0.322	0.231
0–100 USD	43 (41.7)	7 (31.8)	36 (44.4)			
101–200 USD	23 (2.3)	5 (21.7)	18 (78.3)			
201–300 USD	9 (8.7)	2 (22.2)	7 (77.8)			
301–400 USD	3 (2.9)	2 (66.7)	1 (33.3)			
401–500 USD	8 (7.8)	1 (12.5)	7 (87.5)			
501–600 USD	7 (6.8)	1 (14.3)	7 (87.5)			
601 USD or more	10 (9.7)	1 (14.3)	6 (85.7)			
Primary working posture				0.723	0.697	0.070
Seated	6 (5.8)	1 (16.7)	5 (83.3)			
Standing	82 (79.6)	18 (22.0)	64 (78.0)			
Crouching	12 (11.7)	2 (16.7)	10 (83.3)			
Lifting	3 (2.9)	1 (33.3)	2 (66.7)			
Smoker				2.574	0.109	0.158
Never	87 (84.5)	21 (24.1)	66 (75.9)			
Ever	16 (15.5)	1 (6.3)	15 (93.8)			
Alcohol				0.061	0.805	0.024
Never, not currently	26 (25.2)	6 (23.1)	20 (76.9)			
Yes, currently	77 (74.8)	16 (20.8)	61 (79.2)			
Coffee				2.010	0.153	0.140
Never, not currently	7 (6.8)	0 (0.0)	7 9100.0)			
Yes, currently	96 (93.2)	22 (22.9)	74 (71.8)			
BMI				4.345	0.114	0.145
Underweight	17 (17.0)	6 (35.3)	11 (64.7)			
Normal Weight	66 (66.0)	15 (22.7)	51 (77.3)			
Overweight	17 (17.0)	1 (5.9)	16 (94.1)			
No. Meals/Day				0.723	0.697	0.059
3 or fewer	78 (75.7)	18 (23.1)	60 (74.1)			
4	24 (23.3)	4 (16.7)	20 (83.3)			
5 or more	1 (1.0)	0 (0.0)	1 (100.0)			
Meals at same time daily				0.215	0.643	0.046
Yes	56 (54.4)	11 (19.6)	45 (80.4)			
No	47 (45.6)	11 (23.4)	36 (76.6)			
Overall health self-report				18.755	**<0.001**	**0.246**
Very good	6 (5.8)	4 (66.7)	2 (33.3)			
Good	34 (33.0)	12 (35.3)	22 (64.7)			
Acceptable	38 (36.9)	6 (15.8)	32 (84.2)			
Poor	25 (24.3)	0 (0.0)	25 (100.0)			
Overall mood self-report				6.227	**0.043**	**0.174**
Good	40 (38.8)	13 (32.5)	27 (67.5)			
Normal	53 (51.5)	9 (17.0)	44 (83.0)			
Poor	10 (9.7)	0 (0.0)	10 (100.0)			

NOTE: *p*-values in bold indicate a statistically significant association. For all associations where cell counts were less than 5, a Fisher’s exact test was run. All Fisher exact tests failed to find associations. Totals may not add to 103/100% if a participant declined to answer a question or to had a measure taken. n.c. = Cramer’s *v* not calculated because more than half of cells were empty.

**Table 3:** Binary logistic regression of predictor variables to experiencing lower back pain.

Predictor Variable	B	SE	Wald X^2^	*p*	Odds Ratio	95% CI Lower	95% CI Upper
Education							
Primary			4.234	0.120			
Secondary	−1.219	0.596	4.190	0.041	0.295	0.092	0.950
Post-secondary	−0.515	1.064	0.234	0.639	0.598	0.074	4.813
Overall health self-report *							
Poor			5.599	0133			
Acceptable	−21.249	7524.516	0.000	0.998	0.000	0.000	--
Good	−19.721	7524.516	0.000	0.998	0.000	0.000	--
Very good	−18.854	7524.516	0.000	0.998	0.000	0.000	--
Overall, mood self-report *							
Poor			0.052	0.974			
Normal	−17.439	10685.517	0.000	0.999	0.000	0.000	--
Good	−17.302	10685.517	0.000	0.999	0.000	0.000	--

NOTE: * Presence/absence of LBP becomes invariant with presence/absence of poor self-reported health and mood.

## Data Availability

The raw data supporting the conclusions of this article will be made available by the authors on request.
